# Factors Affecting the Metabolic Conversion of Ciprofloxacin and Exposure to Its Main Active Metabolites in Critically Ill Patients: Population Pharmacokinetic Analysis of Desethylene Ciprofloxacin

**DOI:** 10.3390/pharmaceutics14081627

**Published:** 2022-08-04

**Authors:** Martin Šíma, Daniel Bobek, Petra Cihlářová, Pavel Ryšánek, Jaroslava Roušarová, Jan Beroušek, Martin Kuchař, Tomáš Vymazal, Ondřej Slanař

**Affiliations:** 1 Department of Pharmacology, First Faculty of Medicine, Charles University and General University Hospital, 128 00 Prague, Czech Republic; 2 Forensic Laboratory of Biologically Active Substances, Department of Chemistry of Natural Compounds, University of Chemistry and Technology, 166 28 Prague, Czech Republic; 3 Department of Anesthesiology and ICM, Second Faculty of Medicine, Charles University and Motol University Hospital, 150 06 Prague, Czech Republic

**Keywords:** desethylene ciprofloxacin, oxociprofloxacin, formyl ciprofloxacin, population pharmacokinetics, pharmacogenetics, gene polymorphism

## Abstract

The objective of this prospective study was to examine the exposure to the main active metabolites of ciprofloxacin in critically ill patients and to examine the factors (demographic, laboratory and genetic) that could potentially affect the drug metabolic conversion of ciprofloxacin. The secondary aim was to develop a population pharmacokinetic model for the metabolite showing the most associations with the abovementioned factors. A total of 29 patients were treated with intravenous infusion of ciprofloxacin and enrolled on this trial. Blood samples for pharmacokinetic analysis were taken at 1, 4, and 11.5 h following the completion of the infusion. Sex, age, body weight, height, serum creatinine and bilirubin levels, and creatinine clearance (CL_CR_) were recorded, and polymorphisms rs2032582 and rs1045642 in the *ABCB1* gene, rs4148977 in the *SLCO1A2* gene and rs762551 in the *CYP1A2* gene were analyzed. A three-stage parent drug–metabolite population pharmacokinetic model was developed. Median (IQR) metabolite/parent ratios of the desethylene ciprofloxacin, formyl ciprofloxacin and oxociprofloxacin were 5.86 (4.09–9.87)%, 4.08 (3.38–6.92)% and 5.91 (3.42–13.65)%, respectively. The desethylene ciprofloxacin metabolic ratio was positively associated with height (r^2^ = 0.2277, *p* = 0.0089) and CL_CR_ (r^2^ = 0.2023, *p* = 0.0144) and negatively associated with age (r^2^ = 0.2227, *p* = 0.0112). Males had a significantly higher oxociprofloxacin metabolic ratio than females (9.14 vs 3.42%, *p* = 0.0043). In the desethylene ciprofloxacin population PK model, the volume of distribution decreased with age, the parent drug-metabolite transfer rate constant increased with CL_CR_, and the metabolite elimination rate constant decreased with age and is increased in *CYP1A2* rs762551 variant allele carriers. We therefore hypothesized that the CYP1A2 inhibition by ciprofloxacin is mediated by its metabolite desethylene ciprofloxacin.

## 1. Introduction

Ciprofloxacin is a second-generation quinolone antibiotic with efficacy on a broad spectrum of gram-negative and gram-positive bacteria. It is used alone or in combination to treat various severe and life-threatening infections [1,2].

After its oral or intravenous administration, ciprofloxacin is widely distributed to various body tissues and fluids [3,4]. High concentrations of ciprofloxacin are achieved in the kidneys, prostate, liver, lungs, and the urinary and gynecologic tracts, while ciprofloxacin penetration into the central nervous system is limited [4,5]. The majority of the ciprofloxacin dose is excreted unchanged (approximately 60% in the urine and 15% in the feces), while only about 10–15% is eliminated as metabolites [3,6]. Four main metabolites of ciprofloxacin have been identified, namely, desethylene ciprofloxacin, sulfociprofloxacin, oxociprofloxacin and formyl ciprofloxacin [6,7]. The activity of sulfociprofloxacin is negligible. Desethylene ciprofloxacin has an antibacterial activity comparable to that of nalidixic acid. Oxociprofloxacin is less active than ciprofloxacin or norfloxacin, and the activity of formyl ciprofloxacin is for *Escherichia coli* or *Klebsiella pneumoniae* in the range of norfloxacin [8]. Although ciprofloxacin metabolites were identified and chemically characterized many years ago, the metabolic pathways have not yet been clearly described. Similarly, although it is generally well known that various factors, e.g., genetic factors, age, diseases or interacting substances, can significantly influence the metabolic conversion of drugs, the factors influencing the metabolic conversion of ciprofloxacin have not yet been fully studied. Ciprofloxacin is considered a potent inhibitor as well as a substrate of CYP1A2 [9]; however, there is no direct evidence indicating the metabolic pathways, which contribute to the fate of the drug in the body. Ciprofloxacin is a known substrate of P-glycoprotein (ABCB1) and OATP1A2, and these transporters play an important role in its transport across the biological membranes. Therefore, factors affecting its activity (e.g., genetic polymorphisms or inducers/inhibitors) could affect the disposition of ciprofloxacin [10,11].

Therefore, the aim of this study was to examine the exposure to the main active metabolites of ciprofloxacin in critically ill patients and to examine the factors (demographic, laboratory and genetic) that could potentially affect the drug metabolic conversion of ciprofloxacin. The secondary aim was to develop a population pharmacokinetic model for the metabolite showing the most associations with the abovementioned factors.

## 2. Materials and Methods

### 2.1. Study Design

A laboratory blinded, low-intervention prospective pharmacokinetic trial was performed using adult patients who were receiving intravenously administered ciprofloxacin, admitted to the Department of Anesthesiology and Intensive Care Medicine, Second Faculty of Medicine, at Charles University and Motol University Hospital between February 2019 and June 2020. The study followed the requirements of the Declaration of Helsinki and the approval of this study by the Ethics Committee was obtained under the No. EK 1492/18 on 2 January 2019. Written informed consent from all subjects was obtained before enrolment. The study EudraCT registration No. is 2019-003732-24.

Ciprofloxacin was administered as a part of routine medical care according to an approved regimen of 400 or 600 mg every 12 h via intravenous infusion. The prescribed dosing schedule was fully within the competence of the physician. Blood samples for the measurement of ciprofloxacin and its metabolite levels were collected at 1, 4 and 11.5 h following the end of the infusion. Blood samples (5 mL) were collected into serum-collecting tubes without clot activator and were immediately placed in the cold. Samples were then centrifuged at 4500× *g* for 10 min at 4 °C, and the aliquots were stored at −80 °C until the time of the analysis. Further, blood samples (5 mL) for genotyping were collected in tubes containing K2EDTA. The samples were frozen and stored at −20 °C until further processing.

The following patient demographic and laboratory characteristics were retrieved: age, height, body weight, sex, smoking status, serum creatinine and bilirubin levels, and measured creatinine clearance (CL_CR_). CL_CR_ was calculated using the following formula: CL_CR_ = U_CR_ × V/S_CR_, where U_CR_ is the urine creatinine level (µmol/L), V is the urinary flow rate (mL/s) during 24 h urine output, and S_CR_ is the serum creatinine level measured using the enzymatic assay (µmol/L) [12].

### 2.2. Bioanalytical Assay

Ciprofloxacin and its metabolites were analyzed using the method described in detail previously [13] and enriched for the MS/MS parameters of the metabolites, as summarized in Table 1. The metabolite standards (desethylene ciprofloxacin hydrochloride, oxociprofloxacin and formyl ciprofloxacin) were purchased from TRC (Toronto, ON, Canada).

Standard stock solutions were prepared using various solvents, i.e., desethylene ciprofloxacin dissolved in water, formyl ciprofloxacin in water/DMSO (50/50, *v*/*v*), oxociprofloxacin in water/acetic acid (50/50, *v*/*v*) and ciprofloxacin in water/acetic acid (60/40, *v*/*v*). The concentration of these solutions was 1 mg/mL and they were stored at −20 °C.

### 2.3. Genotyping

DNA was extracted from whole blood samples using a genomic DNA purification kit (Elisabeth Pharmacon, Brno, Czech Republic), according to the manufacturer’s instructions. After the measurement of the DNA concentrations using a spectrophotometer, the samples were stored at 5 °C until the time of analysis. The allele-specific TaqMan RT-PCR assay was used to genotype rs4148977 in the *SLCO1A2* gene (the gene encoding the OATP1A2 transporter) and rs762551 in the *CYP1A2* gene (Thermo Fisher Scientific Inc., Waltham, MA, USA) on the BioRad CFX Connect^TM^ Real-Time PCR Detection System (Bio Rad Laboratories Inc., Hercules, CA, USA), and the results were analyzed using BioRad CFX Maestro^TM^ software (Hercules, CA, USA). The genotypes of rs2032582 and rs1045642 in *ABCB1* were detected as described previously [14]. The polymorphisms rs2032582 and rs1045642 in the *ABCB1* gene, rs4148977 in the *SLCO1A2* gene, and rs762551 in the *CYP1A2* gene were selected for analysis, as they represent polymorphisms with known functional significance and an allelic frequency of above 30%.

### 2.4. Data Analysis and Statistics

For both ciprofloxacin and its individual metabolites (desethylene ciprofloxacin, formyl ciprofloxacin and oxociprofloxacin), the area under the concentration–time curve from 0 to 12 h (AUC_12_) was calculated using the linear trapezoidal rule in the PKsolver tool for MS Excel 2013 (Microsoft Corporation, Redmond, WA, USA). In order to make a direct comparison between the parent drug and its metabolites, mass concentrations (ng/mL) were converted to molar concentrations (nmol/mL), and molar concentrations were used for further analyses. Subsequently, the metabolite–parent drug ratio of AUC_12_ was calculated for each ciprofloxacin metabolite. The Mann–Whitney U-test and linear regression model were used to evaluate the relationships between the metabolic ratios and the categorical and continuous variables, respectively. The Kruscal–Wallis test was used for the evaluation of the differences between the genotypes. GraphPad Prism software version 8.2.1 (GraphPad Inc., La Jolla, CA, USA) was used for all comparisons, and *p*-levels of less than 0.05 were considered as statistically significant.

### 2.5. Population PK Model

A parent drug–metabolite population pharmacokinetic model was developed for the metabolite that showed the most associations with demographic/laboratory/genetic factors; this was desethylene ciprofloxacin. Ciprofloxacin and desethylene ciprofloxacin serum concentration–time profiles were analyzed using a nonlinear mixed-effects modeling approach. The model parameters were assumed to be log-normally distributed and were estimated by maximum likelihood using the Stochastic Approximation Expectation Maximization (SAEM) algorithm within Monolix Suite software version 2021R1 (Lixoft SAS, Antony, France). The population model was developed in three steps.

*(1) Base model*

With respect to the parent–metabolite model, with one compartment for both the parent drug and the metabolite, the first order elimination of both the parent drug and metabolite and the unidirectional transformation from the parent drug to the metabolite were tested for the structural model. All PK parameters were considered to be log-normally distributed. Several error models (proportional, additive, and combined) were assessed for the residual error model. The most appropriate model was selected based on the minimum objective function value (OFV), adequacy of the goodness-of-fit (GOF) plots, and low relative standard errors (R.S.E.) of the estimated PK parameters.

*(2) Covariate model*

Bodyweight, height, age, serum creatinine and bilirubin levels, and measured CL_CR_ were tested as the continuous covariates (characteristics predictive of inter-individual variability), while sex and genotypes were tested as the categorical covariates. A preliminary graphical assessment and univariate association using Pearson’s correlation test of the effects of covariates on PK estimates was made. The covariates with *p* < 0.05 were considered for the covariate model. Afterwards, a stepwise covariate modelling procedure was performed. For model selection, a decrease in OFV of more than 3.84 points between the nested models (*p* < 0.05) was considered statistically significant, assuming a χ^2^-distribution. Additional criteria for the model selection were reasonably low R.S.E. values of the estimates of the structural model parameters, the physiological plausibility of the obtained parameter values, and the absence of bias in GOF plots.

*(3) Model evaluation*

The model adequacy was evaluated using GOF plots. Observation values were plotted versus individual and population prediction values. The individual-weighted residuals (IWRES) and population-weighted residuals (PWRES) were plotted versus the predicted concentration plots, and the normalized prediction distribution errors (NPDE) were plotted versus the time after the dose to evaluate for randomness around the line of unity. The visual predictive check (VPC) was performed to evaluate the predictive accuracy of the final model. For this, 1000 replicates of the original dataset were simulated using the final model parameter estimates, and the simulated distribution was compared with that from the observed data. The 90% prediction intervals for the 10th, 50th and 90th percentiles of the simulations were calculated from all replicates and presented graphically.

## 3. Results

**Figure 1:**
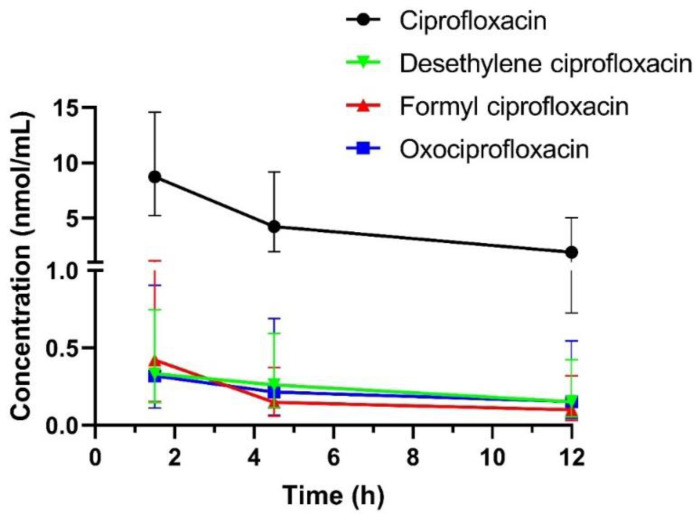
Serum concentration–time profiles of ciprofloxacin and its metabolites. Data are expressed as geometric mean ± SD.

Twenty-nine patients (20 males, 9 females) have been enrolled. Demographic/laboratory characteristics of the patients and the genotype frequencies of *ABCB1 *(rs2032582 and rs1045642), *SLCO1A2 *(rs4148977) and *CYP1A2* (rs762551) are summarized in Table 2. In total, 87 serum concentrations of ciprofloxacin as well as each metabolite (desethylene ciprofloxacin, formyl ciprofloxacin and oxociprofloxacin) were included in the analysis. Geometric mean ± SD serum concentration–time profiles of ciprofloxacin and its metabolites are presented in Figure 1.

The 12-h exposure (AUC_12_) of ciprofloxacin and its metabolites and the metabolite–parent drug ratio for each metabolite are summarized in Table 3. The desethylene ciprofloxacin/ciprofloxacin metabolic ratio was positively associated with height (r^2^ = 0.2277, *p* = 0.0089) and CL_CR_ (r^2^ = 0.2023, *p* = 0.0144), and negatively related to age (r^2^ = 0.2227, *p* = 0.0112). Males had a significantly higher oxociprofloxacin/ciprofloxacin metabolic ratio than females (9.14% vs. 3.42%, *p* = 0.0043). The formyl ciprofloxacin/ciprofloxacin metabolic ratio was associated with none of the characteristics.

For desethylene ciprofloxacin, as the metabolite that showed the most associations with the demographic/laboratory/genetic factors, a parent drug–metabolite population pharmacokinetic model was developed. One compartmental model for both the parent drug and metabolite, with the first order elimination and the unidirectional transformation from the parent drug to the metabolite, was parametrized in terms of the volume of distribution (Vd), parent drug elimination rate constant (K), metabolite elimination rate constant (Km) and the parent drug–metabolite transfer rate constant (Kpm). A proportional error model was the most accurate for the residual and interpatient variability. The population PK estimates for the final model are summarized in Table 4. Among the investigated variables, the most appropriate covariates were age for Vd and Km, CL_CR_ for Kpm and the *CYP1A2* genotype for Km. The final equations describing the relationships between the final model pharmacokinetic parameters and the covariates are following:

Log (Vd)=log (Vd_pop)+β_Vd_age×age+η_Vd

Log (K)=log (K_pop)+η_K

Log (Km)=log (Km_pop)+β_Km_age×age+β_Km_CYP1A2_v×(CYP1A2=v)+η_Km

Log (Kpm)=log (Kpm_pop)+β_Kpm_CLCR×CLCR+η_Kpm

**Figure 2:**
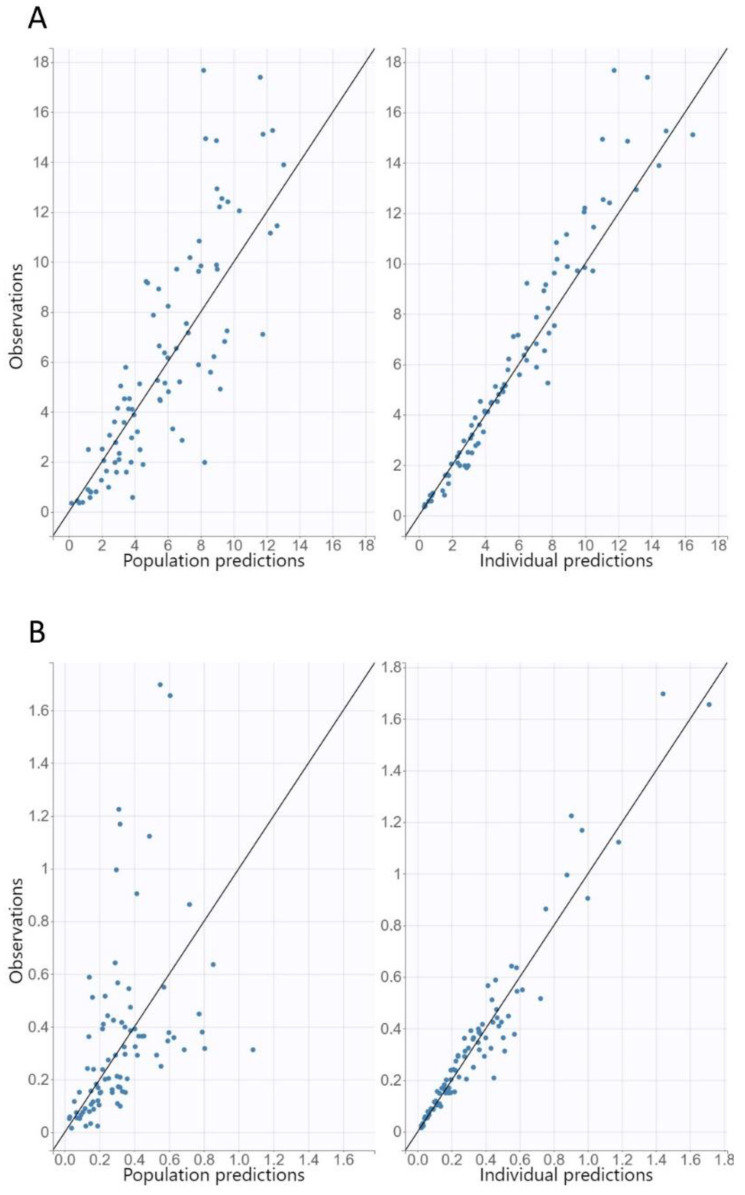
Population and individual predictions of ciprofloxacin (**A**) and desethylene ciprofloxacin (**B**) versus observed concentrations.

**Figure 3:**
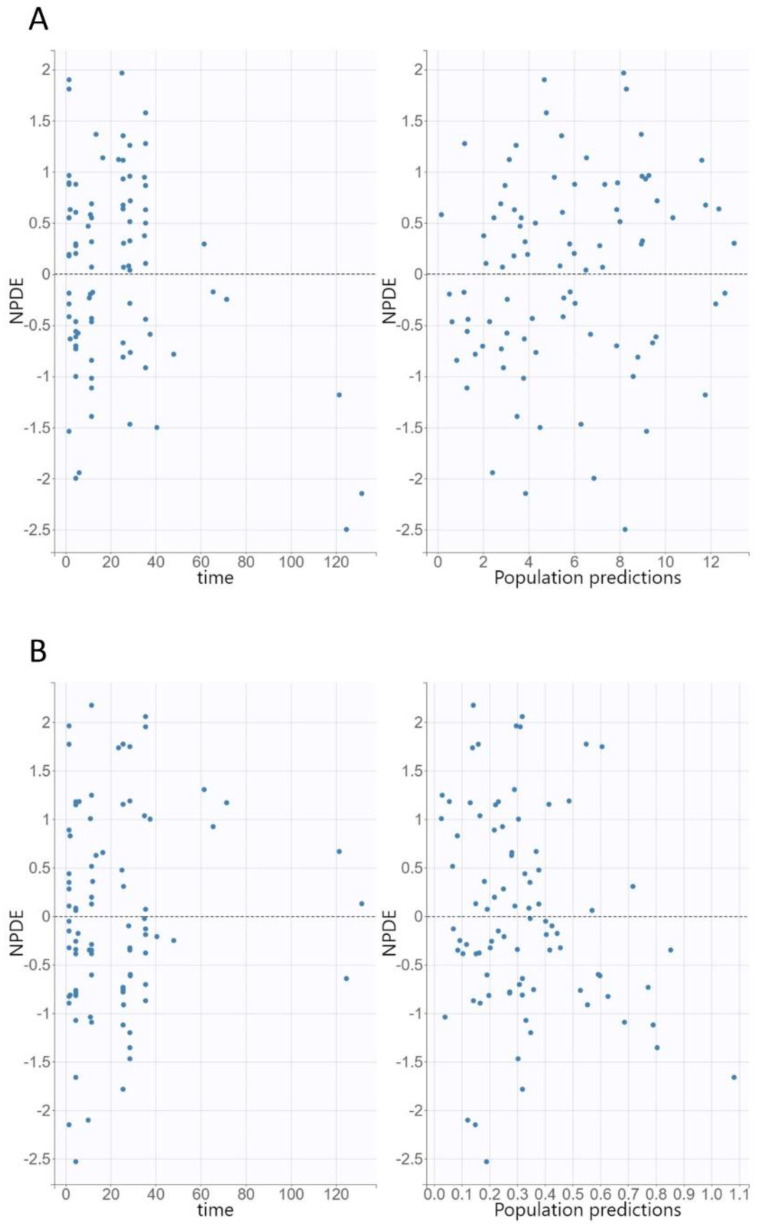
Normalized prediction distribution errors (NPDE) for ciprofloxacin (**A**) and desethylene ciprofloxacin (**B**) versus time and population predictions.

**Figure 4:**
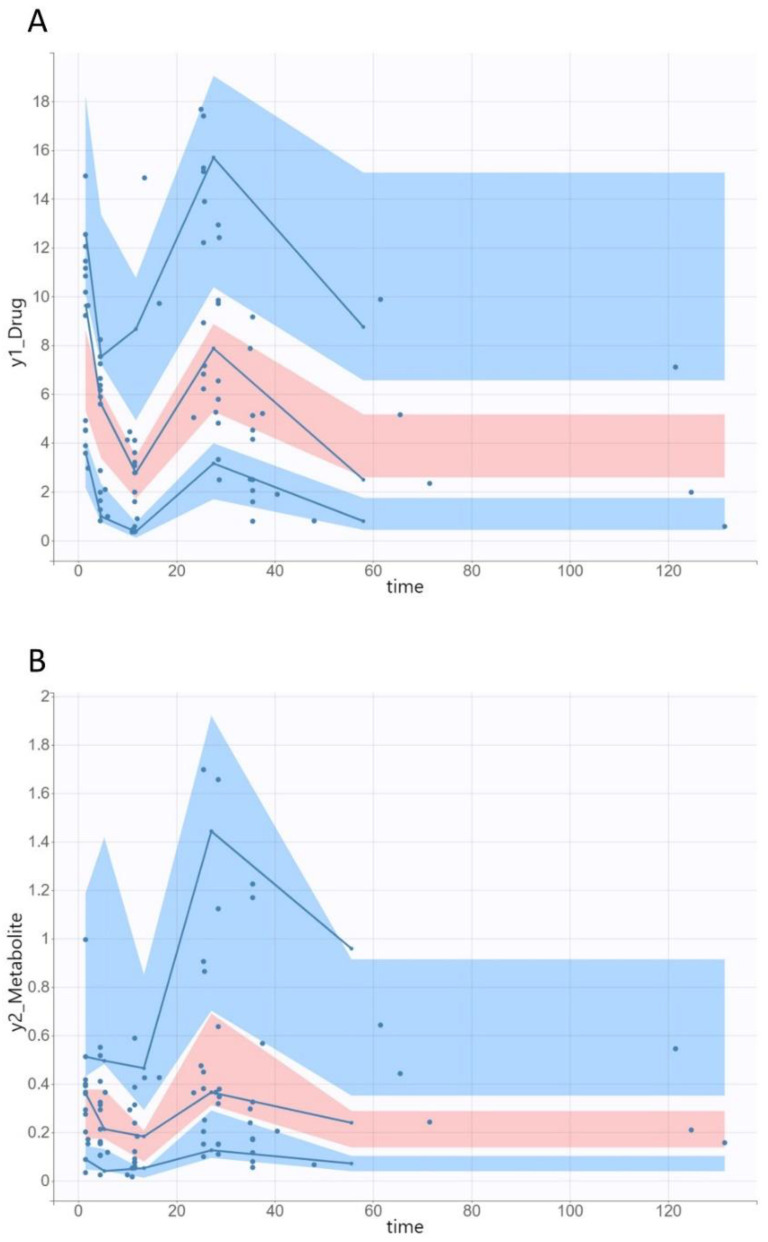
Visual predictive check (shaded areas) and observed data (circles) of ciprofloxacin (**A**) and desethylene ciprofloxacin (**B**) serum concentration versus time for the final model. Solid blue lines represent the 10th, 50th and 90th percentiles of the observed data. Shaded regions represent the 90% confidence interval around the 10th (lower blue region), 50th (pink region) and 90th (upper blue region) percentiles of the simulated data.

The diagnostic GOF plots for both the ciprofloxacin and desethylene ciprofloxacin final covariate models did not show major deviations (Figure 2 and Figure 3). The R.S.E. values revealed that all PK parameters in the population model were precisely estimated. The VPC plots of both the ciprofloxacin and desethylene ciprofloxacin final models revealed that the predictions were consistent with the observations, confirming the validity of the PK model with respect to the concentration–time data (Figure 4).

## 4. Discussion

In this study, we focused on factors affecting the metabolic transformation of ciprofloxacin to its active metabolites in 29 critically ill patients. To our knowledge, this is the largest dataset describing the pharmacokinetics of ciprofloxacin metabolites in this fragile population. If we sum the metabolic ratios for all the metabolites in our study, we obtain a metabolites/ciprofloxacin 12-h exposure ratio of 15.85%. This observation is fully consistent with the data presented in the summary of product characteristics, which states that 61.5% of ciprofloxacin intravenous dose is excreted, unchanged, in the urine, and 15.2% is excreted in the feces, whereas 12.1% of ciprofloxacin is excreted in the urine and feces in the form of metabolites. That means that the proportion of metabolized and unchanged drug is 15.78%. To date, there is very limited knowledge about the factors affecting the metabolic transformation of ciprofloxacin in men. Only the effects of obesity, cirrhosis and renal functions on the pharmacokinetics of ciprofloxacin metabolites have been studied previously [15,16,17]. However, except for the reduced formation of oxociprofloxacin in cirrhotic subjects [16], no remarkable results have been described. We observed no statistically significant covariate for the formyl ciprofloxacin metabolic ratio. On the other hand, ciprofloxacin’s transformation to oxociprofloxacin was almost three times higher in males than in females. Last, but not least, ciprofloxacin’s transformation to desethylene ciprofloxacin increased with height and CL_CR_ and decreased with age. Since both height and CL_CR_ significantly decreased with increasing age (r^2^ = 0.1818, *p* = 0.0211 and r^2^ = 0.5774, *p* < 0.0001, respectively), it can be assumed that only age is a real independent variable for ciprofloxacin’s transformation to desethylene ciprofloxacin. For CL_CR_, as a covariate of the desethylene ciprofloxacin metabolic ratio, we can possibly consider yet another explanation. If ciprofloxacin has a higher potential for renal excretion than its metabolites (and the proportions of ciprofloxacin/its metabolites excreted in the urine/feces as reported by summary of product characteristics suggest this is the case), then in patients with a higher CL_CR_, the parent substance would be excreted more rapidly than its metabolites, and thus the metabolite/parent ratio would increase, as observed in our study and supported by the outputs of the population PK model, in which CL_CR_, as a covariate of Kpm, reduced the unexplained variability in the population PK model much more than age.

Since ciprofloxacin’s metabolic transformation to desethylene ciprofloxacin showed the most associations with the investigated factors, a parent–metabolite population PK model was used to further understand this metabolic transformation and the desethylene ciprofloxacin disposition. Age for Vd and Km, CL_CR_ for Kpm and the *CYP1A2* genotype for Km emerged as the most significant covariates. The volume of distribution and metabolite elimination rate constant decreased with age, the parent drug–metabolite transfer rate constant increased with CL_CR_, and the metabolite elimination rate constant increased in the *CYP1A2* rs762551 variant allele carriers in the population PK model.

The effects of ciprofloxacin on clinically significantly elevated levels of CYP1A2 substrates (e.g., tizanidine, 4-methylaminoantipyrine, clozapine, ropivacaine, theophylline or other xanthine derivatives) have been described extensively in the literature [18,19,20,21,22,23]. Therefore, ciprofloxacin is generally considered as a significant CYP1A2 inhibitor. However, an in vitro study of CYP1A2 human liver microsomes identified only negligible inhibiting activity of ciprofloxacin [24]. This discrepancy, together with our recent observation that the desethylene ciprofloxacin elimination rate constant is associated with the *CYP1A2* genotype, while the parent to metabolite transfer rate constant did not show this relation, indicates that the main inhibitor of the CYP1A2 enzyme is desethylene ciprofloxacin rather than ciprofloxacin. A limitation of the present study is that it provides indirect evidence of the involvement of the ciprofloxacin metabolites in the drug interaction potential. If this hypothesis is directly confirmed in the future, this would mean that the drug interaction potential increases in the elderly, in whom the desethylene ciprofloxacin elimination declines.

## 5. Conclusions

In conclusion, we identified several factors affecting the ciprofloxacin/its active metabolite ratios. Ciprofloxacin’s transformation to oxociprofloxacin is almost three times higher in males than in females. Ciprofloxacin’s transformation to desethylene ciprofloxacin increases with height and CL_CR_ and decreases with age. In the desethylene ciprofloxacin population PK model, the volume of distribution decreases with age, the parent drug–metabolite transfer rate constant increases with CL_CR_, and the metabolite elimination rate constant decreases with age and is increased in *CYP1A2* rs762551 variant allele carriers. We therefore hypothesized that CYP1A2 inhibition by ciprofloxacin is mediated by its metabolite, desethylene ciprofloxacin.

## Figures and Tables

**Table 1:** UHPLC-MS/MS parameters of the analyzed compounds.

	Precursor Ion	Product Ion	Collision Energy	Fragmentor	LOD ^a^	LOQ ^b^
	(*m*/*z*)	(*m*/*z*)	(*V*)	(*V*)	(ng/mL)	(ng/mL)
Ciprofloxacin	332.1	314.1	17	112	9.0	30
		231.0	37			
Desethylene ciprofloxacin	306.1	288.1	13	86	0.9	3.0
		268.1	25			
Formyl ciprofloxacin	360.1	342.1	17	128	0.9	3.0
		215.0	49			
Oxociprofloxacin	346.1	217.0	41	112	0.9	3.0
		286.9	29			
Ciprofloxacin-d8	340.2	322.2	17	100		

^a^—limit of detection (signal-to-noise ratio of 3); ^b^—limit of quantification (signal-to-noise ratio of 10).

**Table 2:** Patient characteristics and genotype frequencies of *ABCB1 *(rs2032582 and rs1045642), *SLCO1A2 *(rs4148977) and *CYP1A2* (rs762551).

Characteristics		Median (IQR)/N (%)
Age (years)		57 (49–71)
Body weight (kg)		90 (70–100)
Height (cm)		175 (168–182)
Serum bilirubin (µmol/L)		11.3 (7.6–17.6)
Serum creatinine (µmol/L)		65 (53–103)
Creatinine clearance (mL/s)		1.29 (0.74–1.91)
*ABCB1* rs2032582	wt/wt	10 (34.5)
	wt/v	16 (55.2)
	*v*/*v*	3 (10.3)
*ABCB1* rs1045642	wt/wt	11 (37.9)
	wt/v	10 (24.5)
	*v*/*v*	8 (27.6)
*SLCO1A2* rs4148977	wt/wt	4 (13.8)
	wt/v	14 (48.3)
	*v*/*v*	11 (37.9)
*CYP1A2* rs762551	wt/wt	6 (20.7)
	wt/v	19 (65.5)
	*v*/*v*	4 (13.8)

**Table 3:** Ciprofloxacin, desethylene ciprofloxacin, formyl ciprofloxacin and oxociprofloxacin: 12-h exposure (AUC_12_) and metabolite–parent drug ratios.

	AUC_12_ (nmol·h/mL)	Metabolite–Parent Ratio (%)
Ciprofloxacin	61.89 (32.14–80.49)	NA
Desethylene ciprofloxacin	3.30 (1.72–4.69)	5.86 (4.09–9.87)
Formyl ciprofloxacin	2.44 (1.27–4.43)	4.08 (3.38–6.92)
Oxociprofloxacin	3.06 (1.53–6.03)	5.91 (3.42–13.65)

Data are expressed as median (IQR). NA: not applicable.

**Table 4:** Estimates of the final ciprofloxacin–desethylene ciprofloxacin population pharmacokinetic model.

Parameter	Estimate	R.S.E. (%)
**Fixed Effects**		
Vd_pop (L)	565.62	25.7
β_Vd_age	−0.022	20.5
K_pop (h^−1^)	0.07	21.3
Km_pop (h^−1^)	3.81	34.6
β_Km_age	−0.035	18.3
β_Km_*CYP1A2*_v	0.6	34.5
Kpm_pop (h^−1^)	0.017	30.1
β_Kpm_CL_CR_	0.81	19.2
**Standard deviation of the random effects**		
Ω_Vd	0.29	16.7
Ω_K	0.24	35.7
Ω_Km	0.31	30.3
Ω_Kpm	0.59	16.6
**Error model parameters**		
b1_parent drug	0.22	11.0
b2_metabolite	0.25	11.5

Vd is volume of distribution; K is parent drug elimination rate constant; Km is metabolite elimination rate constant; Kpm is parent drug–metabolite transfer rate constant; CL_CR_ is creatinine clearance; *CYP1A2*_v is at least one variant allele in the *CYP1A2* genotype.

## Data Availability

The data that support the findings of this study are available from the corresponding author upon reasonable request.
